# Heat-induced phytohormone changes are associated with disrupted early reproductive development and reduced yield in rice

**DOI:** 10.1038/srep34978

**Published:** 2016-10-07

**Authors:** Chao Wu, Kehui Cui, Wencheng Wang, Qian Li, Shah Fahad, Qiuqian Hu, Jianliang Huang, Lixiao Nie, Shaobing Peng

**Affiliations:** 1 National Key Laboratory of Crop Genetic Improvement, Huazhong Agricultural University, Wuhan, Hubei 430070, China; 2 MOA Key Laboratory of Crop Ecophysiology and Farming System in the Middle Reaches of the Yangtze River, College of Plant Science and Technology, Huazhong Agricultural University, Wuhan, Hubei 430070, China; 3 Hubei Collaborative Innovation for Grain Industry, Yangtze University, Jingzhou, Hubei 434023, China

## Abstract

Heat stress causes morphological and physiological changes and reduces crop yield in rice (*Oryza sativa*). To investigate changes in phytohormones and their relationships with yield and other attributes under heat stress, four rice varieties (Nagina22, Huanghuazhan, Liangyoupeijiu, and Shanyou 63) were grown in pots and subjected to three high temperature treatments plus control in temperature-controlled greenhouses for 15 d during the early reproductive phase. Yield reductions in Nagina22, Huanghuazhan, and Liangyoupeijiu were attributed to reductions in spikelet fertility, spikelets per panicle, and grain weight. The adverse effects of high temperature were alleviated by application of exogenous 6-benzylaminopurine (6-BA) in the heat-susceptible Liangyoupeijiu. High temperature stress reduced active cytokinins, gibberellin A_1_ (GA_1_), and indole-3-acetic acid (IAA), but increased abscisic acid (ABA) and bound cytokinins in young panicles. Correlation analyses and application of exogenous 6-BA revealed that high temperature-induced cytokinin changes may regulate yield components by modulating the differentiation and degradation of branches and spikelets, panicle exsertion, pollen vigor, anther dehiscence, and grain size. Heat-tolerant Shanyou 63 displayed minor changes in phytohormones, panicle formation, and grain yield under high temperature compared with those of the other three varieties. These results suggest that phytohormone changes are closely associated with yield formation, and a small reduction or stability in phytohormone content is required to avoid large yield losses under heat stress.

Global climate change analyses predict that the mean air temperature will increase by 1–3.7 °C at the end of the 21st century compared with the mean air temperature during 1850–1900^1^. High temperature extremes on daily and seasonal time scales may occur more frequently in the near future, especially in tropical and subtropical areas where rice (*Oryza sativa*) is widely cultivated^2^.

Rice plants are susceptible to high temperature stress during the reproductive stage^3^,^4^. Grain yield is significantly reduced when air temperature exceeds the critical value for rice growth, especially during highly susceptible growth stages. Exposure to temperatures greater than 33.7 °C for 1 h during anthesis causes spikelet sterility^5^. Exposure to temperatures greater than 38 °C for 8 h can essentially obliterate the rice harvest^6^. Increases in nighttime temperatures also reduced grain yield; nighttime temperatures greater than 27.8 °C during panicle initiation significantly reduced the number of spikelets per panicle and rice yield^7^.

During the early reproductive phase of rice plants, sink characters (i.e., spikelets per panicle and grain size) are determined, which are associated with branch/floret development and growth^8^,^9^. During the middle reproductive phase, panicles undergo exsertion and pollination occurs, which are crucial for spikelet fertility^10^. During the late reproductive phase, non-structural carbohydrates stored in the stems and current assimilates in functional leaves are transported to the panicle and largely accumulate in developing grains^9^,^11^. High temperature during the middle/late reproductive phases reduces spikelet fertility, grain filling, and grain weight, and thus results in low grain yield; however, few studies have investigated the effects of high temperature on yield attributes in different rice varieties during the early reproductive phase^6^,^12^,^13^,^14^.

The formation of different organs in the panicle (including florets, branches, and rhachis) during the early reproductive phase occurs sequentially, and is associated with grain size, panicle elongation, and the differentiation and degradation of branches and florets^8^,^10^. Previous studies report that abiotic stresses such as drought, salinity, and chilling adversely affect panicle formation and cause physiological changes in phytohormone levels and photosynthetic activity, which reduces rice yield components^15^,^16^,^17^,^18^. In other crops such as pearl millet, oats, and sunflower, high temperature during the early reproductive phase also retards panicle development and growth and reduces grain number, seed set, and grain weight^19^,^20^,^21^. Spikelet sterility and yield loss have been observed in rice varieties in response to high temperature during the early reproductive phase^22^,^23^. Morphological characters of the lemma and palea also change significantly in response to high temperature during the early reproductive phase^24^. However, Jagadish *et al*.^3^ reported that high temperature during panicle initiation has neglectable effects on spikelet number and spikelet fertility in rice varieties. The results of these studies indicate that the understanding of high temperature effects on morphological and physiological traits and yield attributes is far from complete.

Phytohormones regulate aerial organ formation, panicle characters, and seed yield^25^,^26^,^27^. Auxins (IAA), gibberellins (GAs), and cytokinins (CTKs) act as central regulators that modulate organogenesis and panicle formation by regulating cell proliferation and expansion in the shoot meristem^25^,^26^,^28^. For example, IAA and GA_1_ stimulate internode elongation and panicle exsertion at heading, whereas reduced levels of IAA and GA_1_ induce panicle enclosure and spikelet sterility in rice and barley^29^,^30^. Fluctuations in CTK levels lead to changes in branch and floret numbers, which affects panicle size in rice^27^,^31^.

Phytohormones are involved in plant responses to abiotic stresses such as high temperature^4^,^32^,^33^,^34^. High temperature modulates phytohormone levels, thereby affecting plant organ growth and yield. For example, high temperature increased abscisic acid (ABA) levels and reduced IAA and GA levels in anthers and developing grains of rice, thereby reducing spikelet fertility and grain weight^35^,^36^. In passion fruit, high temperature attenuated shoot meristem development and caused flower abortion by lowering the CTK levels, and CTK treatment protected the developing floral meristems against high temperature stress^37^. However, little is known about the effects of high temperature on phytohormones in young panicles and possible effects on panicle development and yield attributes in rice.

Rice varieties display considerable genotypic variations in tolerance to high temperature stress^23^. This may explain differences in high temperature effects on yield components during the early reproductive phase in rice^3^,^22^,^23^. Therefore, we investigated genotypic variations in phytohormone responses to high temperature treatments and their relationship with panicle characters and yield attributes in rice. Our goal was to elucidate the pathways involved in yield loss due to high temperature conditions during the early reproductive phase.

## Results

### High temperature treatments

Mean daytime temperature under high daytime temperature (HDT) treatment was 36.1 °C, which was approximately 4.2 °C higher than that of the control. Mean nighttime temperature under high nighttime temperature (HNT) treatment was 31.9 °C, which was approximately 4.7 °C higher than that of the control. Mean daytime and nighttime temperatures under high whole-day temperature (ADT) treatment were 38.3 and 31.5 °C, which were approximately 6.4 and 4.3 °C higher than those of the control, respectively (Table 1).

### Effects of high temperature treatments on grain yield and yield components

**Figure 1:**
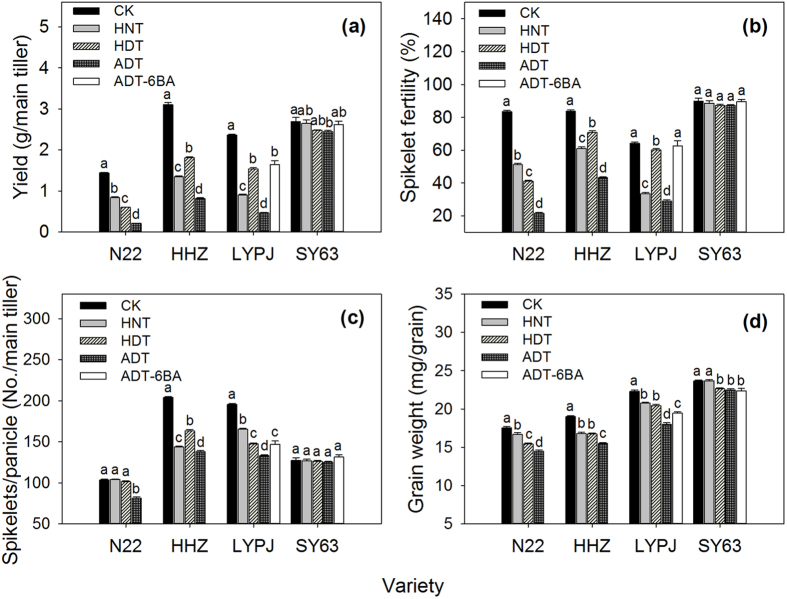
Effects of high temperature treatments on yield and yield components. Data are presented as mean ± SD (n = 4). Different letters indicate significant differences among the four different temperature treatments for the same variety at the *P* < 0.05 level by a least significant difference (LSD) test. ADT, high whole-day temperature treatment; CK, control; HDT, high daytime temperature treatment; HNT, high nighttime temperature treatment.

The three high temperature treatments significantly reduced grain yield compared with those of the controls, especially in the varieties Nagina22 (N22), Huanghuazhan (HHZ), and Liangyoupeijiu (LYPJ) (Fig. 1a). For example, the grain yields under high nighttime temperature treatments were reduced by 41% for N22, 56% for HHZ, 62% for LYPJ, and 1% for Shanyou 63 (SY63); under high daytime temperature treatments, grain yields were reduced by 58% for N22, 42% for HHZ, 35% for LYPJ, and 8% for SY63. The largest reductions in grain yield were observed under ADT in all four varieties, with grain yield reduced by 85% for N22, 74% for HHZ, 80% for LYPJ, and 9% for SY63. The relative yield of SY63 was significantly higher than that of LYPJ, HHZ, and N22 under each high temperature treatment.

The three high temperature treatments significantly reduced spikelet fertility in the varieties N22, HHZ, and LYPJ compared with that in the control (Fig. 1b); however, spikelet fertility in SY63 was only slightly but insignificantly reduced by high temperature treatments. The three high temperature treatments significantly reduced the number of spikelets per panicle in HHZ and LYPJ; however, the three high temperature treatments caused only a small and insignificant reduction in spikelets per panicle in SY63 (Fig. 1c).

The HNT and HDT treatments had no marked effect on the number of spikelets per panicle in N22, whereas ADT significantly reduced the number of spikelets per panicle in N22. Grain weight was reduced by high temperature treatments compared to the control (Fig. 1d), especially under ADT. Generally, N22, HHZ, and LYPJ had relatively large reductions in grain weight under high temperature treatments.

The application of exogenous 6-benzylaminopurine (6-BA), a synthetic cytokinin, significantly increased grain yield, grain weight, spikelet fertility, and spikelets per panicle under ADT by 220, 7.8, 116, and 10.4% in LYPJ, and by 8.3, 0.0, 2.6, and 4.8 in SY63, respectively, compared with the results of ADT without 6-BA application (Fig. 1).

### Effects of high temperature treatments on panicle traits

**Figure 2:**
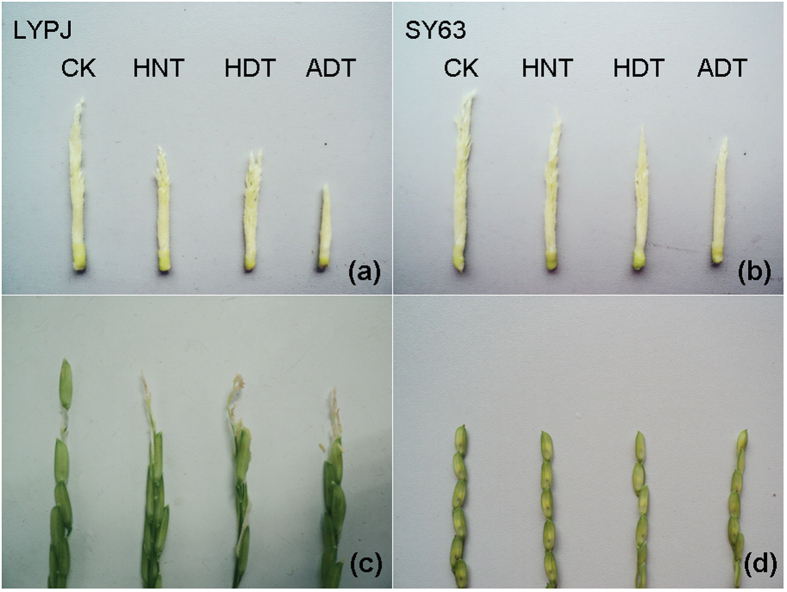
Effects of high temperature treatments on young panicle growth and floret degradation at 10 days after high temperature treatments. (**a**,**b**) indicate young panicles of LYPJ and SY63, (**c**,**d**) indicate floret degradation in LYPJ and SY63. ADT, high whole-day temperature treatment; CK, control; HDT, high daytime temperature treatment; HNT, high nighttime temperature treatment.

The three high temperature treatments had no obvious effects on the numbers of differentiated primary branches (PB) and attached differentiated florets (FPB) in the four rice varieties (Table 2). The three high temperature treatments had no effects on the numbers of secondary branches (SB) and attached florets (FSB) in SY63 and N22, except N22 was negatively affected by ADT; however, high temperature treatments negatively affected SB and FSB in HHZ and LYPJ, especially under ADT. The three high temperature treatments significantly inhibited panicle growth and spikelet differentiation in LYPJ (Fig. 2a), but did not substantially affect those in SY63 (Fig. 2b).

The three high temperature treatments affected the degradation of floret and branches. Overall, the numbers of retrograded secondary branches (RSB) did not increase under the three high temperature treatments in SY63, whereas more RSB were observed in the other three varieties, especially under ADT, which caused RSB increases of 23% in N22, 18% in HHZ, and 60% in LYPJ. High temperature treatments had negligible or small effects on the numbers of retrograded florets on primary branches (RFPB); however, they markedly increased the numbers of retrograded florets on secondary branches (RFSB) in N22, HHZ, and LYPJ (Table 2 and Fig. 2c), especially under ADT, but not in SY63 (Table 2 and Fig. 2d).

**Figure 3:**
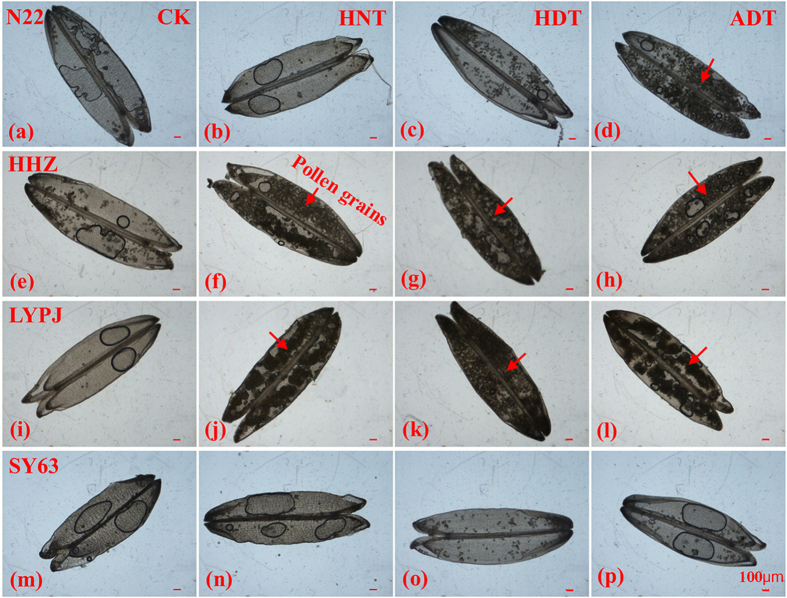
Illustration of pollen grains retained within the anthers under high temperature treatments. (**a**–**d**) N22, (**e**–**h**) HHZ, (**i**–**l**) LYPJ, and (**m**–**p**) SY63. ADT, high whole-day temperature treatment; CK, control; HDT, high daytime temperature treatment; HNT, high nighttime temperature treatment. Scale bar = 100 μm.

**Figure 4:**
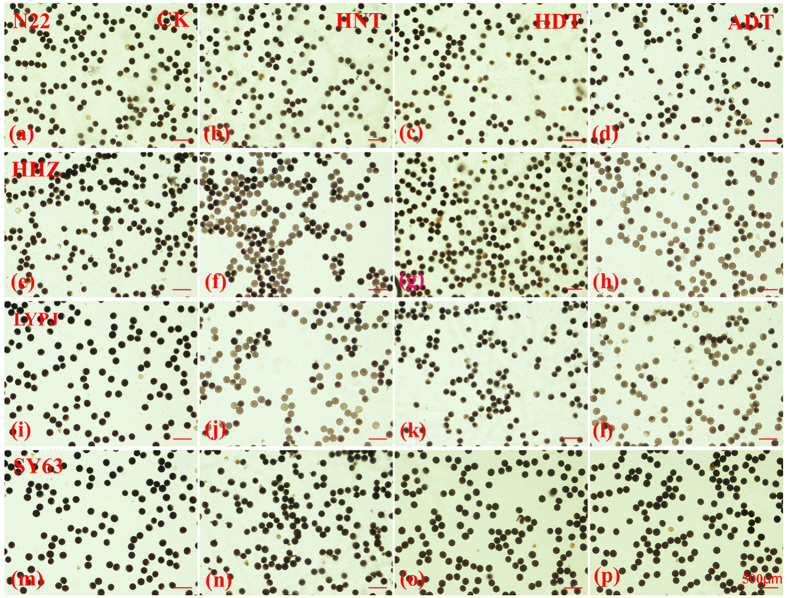
Effects of high temperature treatments on pollen vigor. (**a**–**d**) N22, (**e**–**h**) HHZ, (**i**–**l**) LYPJ, and (**m**–**p**) SY63. ADT, high whole-day temperature treatment; CK, control; HDT, high daytime temperature treatment; HNT, high nighttime temperature treatment. Scale bar = 500 μm.

The three high temperature treatments dramatically reduced the lengths of exserted panicles. This increased the degree of panicle enclosure in N22, HHZ, and LYPJ, especially under ADT. However, panicle enclosure was not observed in SY63. High temperature treatments also disrupted anther dehiscence, which was decreased in HHZ and LYPJ, especially under ADT. We also observed adverse effects on pollen shedding (Fig. 3); the bulk of pollen grains remained adhered within the anthers under the three high temperature treatments in HHZ (Fig. 3f–h) and LYPJ (Fig. 3j–l), and under ADT in N22 (Fig. 3d). However, anther dehiscence and pollen shedding was not significantly affected by high temperature in N22 under HNT and HDT, and in SY63 under the three high temperature treatments. High temperature treatments also adversely affected pollen vigor; the percentages of vigorous pollen grains were decreased in HHZ and LYPJ, but were not significantly decreased in N22 and SY63 (Table 2 and Fig. 4).

Compared with those in the control (CK), grain shape traits (grain length, grain width, and grain area) were significantly decreased in the four varieties under the high temperature treatments (Table 2). HNT had less severe effects on grain shape traits, whereas HDT and ADT had similar and larger adverse effects on grain shape traits.

Under ADT, exogenous 6-BA treatment increased SB, FSB, exserted panicle length, and grain length, and significantly decreased RSB and RFSB in LYPJ, compared with those without exogenous 6-BA treatment. However, 6-BA application to SY63 under ADT did not significantly affect these traits (Table 2).

### Effects of high temperature treatments on phytohormone contents in panicles

Total CTKs were relatively stable in all heat treated and control samples in N22, HHZ, and SY63, whereas the three high temperature treatments significantly reduced total CTKs in LYPJ (Table 3). ADT reduced total CTKs by approximately 9% in N22, 7% in HHZ, 23% in LYPJ, and 7% in SY63. However, active CTKs were significantly reduced by high temperature treatments compared with those in the control. For N22 and SY63, there were no significant differences in active CTKs between HNT and HDT. Generally, the content of active CTKs was the lowest under ADT, and decreased by approximately 55% in N22, 55% in HHZ, 57% in LYPJ, and 16% in SY63.

Generally, the three high temperature treatments showed similar effects on tZ + tZR (tZ, *trans*-zeatin; tZR, *trans*-zeatin riboside) and iP + iPA + iPMP (iP, N^6^-(Δ^2^-isopentenyl) adenine; iPA, N^6^-(Δ^2^-isopentenyl) adenosine riboside; iPMP, isopentenyladenine riboside-5′- monophosphate) as they did on active CTKs. The three high temperature treatments did not significantly affect the contents of tZ + tZR and iP + iPA + iPMP in SY63 according to the relative percentage reduction (Table 3) and compared with the other three varieties. By contrast, the three high temperature treatments significantly enhanced the contents of tZ9G + iP9G (tZ9G, *trans*-zeatin 9-glucoside; iP9G, isopentenyladenine 9-glucoside) in N22, HHZ, and LYPJ, but not in SY63, compared with those of the control.

The three high temperature treatments significantly reduced GA_1_ contents in N22 (45, 50, and 69% reduction under HNT, HDT, and ADT, respectively), HHZ (23, 15, and 50% reduction under HNT, HDT, and ADT, respectively) and LYPJ (29, 11, and 42% reduction under HNT, HDT, and ADT, respectively) compared with the control. However, the heat treatments did not affect GA_1_ contents in SY63 (5, 2, and 0% reduction under HNT, HDT, and ADT, respectively). Similar trends were observed for IAA contents. By contrast, high temperature treatments significantly increased the ABA contents in SY63 (36, 41, and 42% increases under HNT, HDT, and ADT, respectively) and N22 (7, 21, and 25% increases under HNT, HDT, and ADT, respectively), but had less effect on ABA contents in HHZ (14, 14, and 14% increases under HNT, HDT, and ADT, respectively) and LYPJ (11, 12, and 13% increases under HNT, HDT, and ADT, respectively).

### Relationships of changes in panicle characters and changes in phytohormones

As shown in Table 4, the change in inactive CTKs (iP9G + tZ9G) was negatively correlated with changes in grain yield, spikelets per panicle, PB, SB, spikelet fertility, exserted panicle length, pollen vigor, and grain weight, and positively correlated with change in RFSB. The decreases in both tZ + tZR and iP + iPA + iPMP were positively correlated with reductions in grain yield, spikelets per panicle, SB, FSB, grain weight, and grain length, and negatively correlated with increases in RSB and RFSB.

The decreases in active CTKs were positively correlated with decreases in grain yield, spikelets per panicle, SB, FSB, grain weight, and grain length, and negatively correlated with increases in RSB and RFSB. The decreases in total CTKs were positively correlated with reductions in FPB, FSB, grain weight, and grain length, and negatively correlated with increases in RSB and RFSB.

Generally, the decreases in GA_1_ and IAA showed similar positive correlations with reduction in spikelet fertility, exserted panicle length and grain weight. On the other hand, decreases in IAA were positively correlated with reductions in grain yield, FPB, FSB, anther dehiscence, and pollen vigor; however, it was negatively correlated with increases in RSB and RFSB. The changes in ABA were positively correlated with changes in grain yield, spikelets per panicle, and SB (Table 4).

## Discussion

Rice plants are highly vulnerable to high temperature during the reproductive phase. As shown in Table 1, the average daytime temperatures of the high temperature treatments (38.3 °C under ADT, 36.1 °C under HDT, and 33.5 °C under HNT) were approximately 2–7 °C higher than those of the control. The average nighttime temperatures were approximately 32 °C under HNT and ADT, which is 5 °C higher than those of the control. Sánchez *et al*.^38^ reported that the maximum temperature for optimal panicle development in rice is 33.1 °C during panicle initiation. In the present study, the three high temperature treatments during the early reproductive phase reduced rice yields to different extents depending on the rice variety (Fig. 1). The reductions in yield components were associated with adverse effects of high temperature on panicle traits (Table 2). Therefore, the temperature treatments may be considered as high temperature stress for panicle growth.

### Yield reductions under high temperature

**Figure 5:**
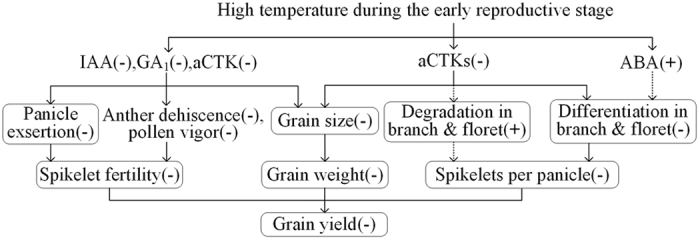
The proposed mechanism for phytohormone-mediated yield reductions in response to high temperature conditions during the early reproductive stage in rice. −and + indicate a decrease and an increase in the trait value under high temperature treatments compared with those in the control, respectively; → indicates positive correlation between two traits; ⤑ indicates negative correlation between two traits; The trait with a box indicates that the adverse effect of high temperature treatments on the trait was relieved by application of exogenous 6-BA. aCTKs, active cytokinin compounds (tZ + tZR + iP + iPA + iPMP).

Rice yield is determined multiplicatively by yield components. As shown in Fig. 1, high temperature treatments significantly reduced spikelet fertility, spikelets per panicle, grain weight, and yield in N22, HHZ, and LYPJ. Similar reductions were observed for grain filling percentage (data not shown). These observations are consistent with previous studies showing that high temperature during the early reproductive phase reduced spikelet fertility and spikelets per panicle in rice^7^,^22^,^23^, and reduced grain number and grain weight in oats and sunflower^19^,^21^. Grain yield and yield components in SY63 were not significantly affected by the three high temperature treatments (Fig. 1), indicating that SY63 is more tolerant to high temperature during the early reproductive phase than the other three varieties. The decrease in grain yield was positively correlated with reduction in spikelet fertility (*r* = 0.58, *n* = 12, *P* < 0.05), reduction in spikelets per panicle (*r* = 0.88, *n* = 12, *P* < 0.01), reduction in grain weight (*r* = 0.85, *n* = 12, *P* < 0.01), and reduction in grain filling percentage (*r* = 0.61, *n* = 12, *P* < 0.05) across the four varieties and the three high temperature treatments. These results indicate that high temperature-induced yield reductions are associated with synchronous reduction in the three yield components under high temperature treatments during the early reproductive phase (Fig. 5).

Jagadish *et al*.^3^ reported that high temperature during the early reproductive phase had no influence on spikelet fertility and spikelets per panicle in rice. Studies evaluating high temperature effects generally consider the plant variety, the intensity/duration of high temperature, and the growth phase subjected to high temperature stress. In our study, SY63 showed tolerance to high temperature during the early reproductive phase, whereas N22, HHZ, and LYPJ were susceptible (Fig. 1). Das *et al*.^23^ reported that there were distinct variations in the responses to high temperature at panicle initiation between lowland and upland rice genotypes. The high temperature injury caused by ADT (combination of HDT and HNT) during the early reproductive phase was more serious than that of HDT or HNT (Fig. 1). At flowering, spikelet fertility decreased linearly as the temperature increased and the duration of high temperature was prolonged^5^,^6^. The accumulated injury caused by exposure to high temperature for four days (09.00–15.00 hr) in the study of Jagadish *et al*.^3^ may be not as serious as the injury caused by exposure for 15 days in the present study. Therefore, these combined results suggest that high temperature stress during the early reproductive phase reduces grain yield to different degrees depending on the plant variety, the high temperature intensity, and the duration of high temperature.

The rice cultivar N22 is considered as highly tolerant to the effects of high temperature^3^,^6^,^39^. However, we observed significant reductions in spikelet fertility, grain yield, and yield components under high temperature treatments during the early reproductive phase, especially under ADT (Fig. 1). In most of the previous studies on N22, high temperature treatments were conducted at flowering and N22 showed normal anther dehiscence, sufficient pollen reception, and high pollen viability, which would maintain high spikelet fertility under high temperature conditions^39^,^40^. These results suggest that N22 may be tolerant to high temperature during flowering but not during the early phase of reproductive development. Shi *et al*.^14^ reported that rice plants show contrasting responses to high temperature stress at anthesis compared with those at gametogenesis. Another study of our group found that N22 showed strong tolerance to heat stress imposed during flowering, whereas it was susceptible to high temperature imposed during panicle formation^41^.

Spikelet sterility in N22 was associated with panicle enclosure caused by high temperature treatments, which was consistent with a previous report that spikelet fertility of N22 decreased significantly due to panicle enclosure caused by 38 °C for 5 days^42^. A recent study found no effects of short-term high temperature (38 °C during 08.30–14.30 hr for 1 or 3 days) on panicle exsertion in N22^39^. These combined results suggest that the resistance/tolerance of N22 to high temperature may depend on both the growth stage and the intensity/duration of high temperature stress conditions.

### Responses of panicle characters to high temperature

Differentiation and degradation of branches/florets determines the number of spikelets per panicle and floret size in rice^10^. In this study, high temperature treatments attenuated differentiation of secondary branches and attached florets, and promoted their degradation in susceptible varieties, especially under ADT. For example, HNT and ADT reduced SB by 15 and 20% in HHZ, and by 11 and 17% in LYPJ (Table 2). In millet and sunflower, high temperature adversely affected panicle differentiation and thus reduced the number of florets^20^,^21^. We observed that the adverse effects of ADT on differentiation of secondary branches and attached florets were alleviated by application of exogenous 6-BA, which resulted in an increase in spikelets per panicle in LYPJ compared with those under ADT without 6-BA application (Table 2). Panicle traits (such as branches, spikelets, and panicle exsertion) in the tolerant SY63 were not significantly affected by high temperature treatments, and produced relatively more spikelets per panicle and higher grain yield under high temperature treatments than the other varieties (Table 2). Significant correlations between decreases in spikelets per panicle and changes in differentiation of branches/florets were identified (*r* = 0.70 with reduction in PB, *P* < 0.05; *r* = 0.97 with reduction in SB, *P* < 0.01; *r* = 0.70 with reduction in FSB, *P* < 0.05; *r* = 0.63 with increase in RSB, *P* < 0.05; *r* = 0.87 with increase in RFSB, *P* < 0.01) across the four varieties and the three high temperature treatments. The results in this study and previous reports indicate that high temperature reduces spikelets per panicle as a consequence of increased degradation and suppressed differentiation of secondary branches and florets.

Reduced elongation of internodes often results in panicle enclosure within the flag leaf sheath, which blocks normal pollination and leads to spikelet sterility^43^. Panicle enclosure in rice can be induced by genetic or environmental factors such as drought, chilling, and insufficient sunlight^10^,^43^,^44^. Recent studies report that high temperature during panicle initiation induces panicle enclosure in rice^22^,^23^; however, those studies did not consider the effect of panicle enclosure on spikelet fertility under high temperature. For example, Das *et al*.^23^ attributed high temperature-induced spikelet sterility only to impaired male fertility (reduced pollen viability, reduced pollen tube growth, impaired anther dehiscence, and fewer pollen landing on stigma). Disruption of stamen function and pistil hypoplasia are both associated with spikelet sterility under high temperature stress during the early reproductive stage^24^.

This study showed that spikelet fertility was substantially reduced in rice varieties that were susceptible to high temperature, and was associated with panicle enclosure in response to high temperature treatments during the early reproductive phase (Table 2 and Fig. 1b). However, panicle enclosure was not induced by high temperature treatments in the tolerant SY63 that produced relatively higher spikelet fertility under high temperature treatments (Table 2). Although the percentage of sterility due to incomplete panicle exsertion and high temperature stress was not determined, there was close relationship between the decrease in exserted panicle length and reduction in spikelet fertility (*r* = 0.87, *n* = 12, *P* < 0.01). In heat-susceptible LYPJ, application of exogenous 6-BA attenuated heat-induced panicle enclosure (Table 2) and increased spikelet fertility under ADT (Fig. 1b). Pollen vigor and anther dehiscence were decreased in heat-susceptible HHZ and LYPJ in our study (Figs 3 and 4). Shah *et al*.^45^ also reported adverse effects of heat stress on pollen vigor and anther dehiscence under high temperature regimes during the early reproductive phase in HHZ and IR64. High temperature treatment may disrupt the function of septum and tapetum, and delayed locule opening in rice^46^. In barley, high temperature during panicle differentiation resulted in pollen grains with severely reduced cytoplasm, which caused high spikelet sterility^47^. These combined results suggest that spikelet sterility may be associated with both panicle enclosure and male sterility under high temperature treatments during the early reproductive phase (Fig. 5).

Grain weight was reduced markedly under high temperature treatments (Fig. 1). A modeling analysis revealed that temperature during panicle initiation acts as a main environmental determinant of rice grain weight^48^. Rice grain weight is determined by grain size (length, width, and thickness) and grain plumpness^10^. In this study, high temperature treatments caused heat-susceptible varieties to produce smaller grains (Table 2), but had relatively less effect on grain size and grain weight in heat-tolerant SY63 (Table 2 and Fig. 1). In heat-susceptible LYPJ, exogenous 6-BA application under ADT induced increases in grain length and grain weight (Table 2 and Fig. 1d). We found that reduction in grain weight was positively correlated with reduction in grain area (*r* = 0.60, *n* = 12, *P* < 0.05) and reduction in grain length (*r* = 0.72, *n* = 12, *P* < 0.01). Grain size in rice is largely associated with the glume shape, which is determined during panicle development^9^,^21^. The heat-induced decrease in grain size may be partially explained by the fact that the development and morphology of the lemma and palea are changed considerably by high temperature^24^. Therefore, high temperature during the early reproductive phase may adversely affect grain weight via grain size. Non-structural carbohydrates in stems and sheaths, current assimilates in functional leaves, and their translocation through vascular bundles also are associated with final grain weight in rice^9^,^11^. High temperature reduces the abundance of non-structural carbohydrates in stems^13^ and hinders vascular bundle development^49^. Based on the previous findings, high temperature during the early reproductive phase may reduce grain weight by influencing grain filling in heat-sensitive rice plants.

### Effects of high temperature on phytohormone levels and panicle formation

High temperature treatment during the early reproductive phase reduced the active CTKs and increased the CTKs conjugated with glucose (tZ9G and iP9G) in the panicles of heat-susceptible rice varieties (Table 3). In *Phalaenopsis* leaves, high temperature also reduced active CTKs and led to the accumulation of glucoside-type CTKs^50^. The reduction in active CTKs may be attributed to enhanced degradation by heat-activated CTK oxidase/dehydrogenase^33^. We found that the changes in tZ + tZR, iP + iPA + iPMP, active CTKs, and total CTKs were closely associated with the retrograded branches and florets, the differentiated branches and florets and spikelets per panicle under three high temperature treatments (Tables 2, 3, 4). In heat-susceptible LYPJ, an increase in differentiated branches and florets were induced by exogenous 6-BA treatment, and more spikelets per panicle were recorded under ADT (Fig. 1c). Similarly, heat-induced reductions in CTKs disrupted floral primordial development and led to flower abortion in passion fruit, whereas application of 6-BA protected the developing floral primordia^37^. Additionally, exogenous 6-BA application increased the tZ type CTKs in rice tiller buds^51^. In rice, enhanced degradation of CTKs in meristems resulted in fewer spikelets^31^, whereas enhanced local synthesis of CTKs stimulated the production of more spikelets per panicle^27^. Therefore, heat-induced fluctuations in CTKs in rice may affect the differentiation and degradation of branches and florets, and further affect the spikelets per panicle. Compared with the heat-sensitive varieties, the heat-tolerant SY63 had only minor reductions in active CTKs under high temperature treatments, and had higher relative spikelets per panicle (Table 3). These combined results suggest that CTK stability was involved in heat tolerance responses, and played a role in maintaining higher relative panicle size under heat stress.

High temperature treatments increased ABA levels in our study (Table 3). Increases in ABA content are frequently induced by abiotic stresses^52^. High temperature up-regulated ABA biosynthesis-related genes and down-regulated ABA catabolism-related genes^32^. The increase in ABA was associated with modestly higher grain yield and spikelets per panicle under high temperature treatments (Table 4). The highest increase in ABA was observed in heat-tolerant SY63, which had higher grain yield and related components under high temperature treatments compared with those of the other three varieties (Table 3). A high level of endogenous ABA is often responsible for tolerance to heat stress, and ABA treatment alleviates the adverse effects of heat stress^53^. These combined results suggest that increases in ABA and stability in active CTKs may avoid large reductions in grain yield and spikelets per panicle under high temperature treatments by maintaining differentiation and reducing the degradation of branches and florets (Fig. 5).

High temperature treatments reduced IAA and GA_1_ contents in young panicles of heat-susceptible varieties (Table 3). Heat-induced reductions in IAA and GA_1_ also were reported in anthers and developing grains in rice^35^,^36^. High temperature generally suppresses IAA and GA biosynthesis^32^,^54^. GA_1_ may act as mobile signal in plants^55^, and is associated with plant height and internode elongation in rice^56^. Panicle-derived IAA regulates GA_1_ biosynthesis in the internode, which is essential for shoot elongation and panicle exsertion in cereals^29^,^30^. We observed that decreased GA_1_ correlated with reductions in exserted panicle length and spikelet fertility, and decreased IAA correlated with reductions in exserted panicle length, anther dehiscence, pollen vigor, and spikelet fertility (Table 4). IAA and GA_1_ levels were influenced slightly by high temperature treatments in the heat-tolerant SY63 that displayed complete panicle exsertion, relatively high pollen vigor, relatively high anther dehiscence, and relatively high spikelet fertility under heat treatments (Table 3). Similarly, Sakata *et al*.^54^ reported that IAA regulated pollen development and male sterility under high temperature. Exogenous 6-BA application promoted complete panicle exsertion and increased spikelet fertility in LYPJ under ADT (Table 2 and Fig. 1b). In pea plants, exogenous 6-BA application to the shoot apex increased local IAA synthesis, promoted IAA export from the treated apex and basipetal transport in stems, and further regulated shoot growth^57^. These results suggest a possible mechanism for low spikelet fertility under high temperature, in which heat inhibits panicle exsertion and induces male sterility by reducing the levels of active CTKs, IAA, and GA_1_ (Fig. 5).

Decreases in the contents of IAA, GA_1_, tZ + tZR, and iP + iPA + iPMP were closely correlated with the reductions in grain size (grain length) and grain weight (Table 4). Grain size is related to glume development, which is regulated by CTKs, IAA, and GAs via cell division and enlargement^26^,^28^. Under field conditions, IAA, GA_3_, and CTK treatments improved the growth and development of rice florets^25^. Heat stress may reduce IAA, GA_1_, and CTKs, which results in small grain size and low grain weight in susceptible varieties (Fig. 5). In our study, exogenous 6-BA treatment increased grain length and grain weight in LYPJ under ADT (Table 2 and Fig. 1d). IAA, GA_1_, and CTKs were slightly affected by high temperature treatments in the heat-tolerant SY63, which had higher relative grain weight and grain size than heat-susceptible varieties (Tables 2, 3 and Fig. 1d), indicating that the stability of IAA, GA_1_, and CTKs may be involved in improved grain weight and grain size in heat-tolerant varieties under high temperature conditions (Fig. 5).

It is noteworthy that roles of CTKs on panicle exsertion, spikelets per panicle, grain weight and grain yield under high temperature treatments were supported by both correlation analyses and application of exogenous 6-BA in the study, however, effects of IAA, GA_1_, and ABA on the investigated traits under high temperature treatments were only supported by correlation analyses. Therefore, investigations on the effects of exogenous IAA, GA_1_, and ABA applications are needed to confirm their roles on panicle formation and grain formation under high temperature treatments.

## Conclusions

High temperature treatments during the early reproductive phase of rice reduced yield and yield components (Fig. 1), increased the numbers of retrograde secondary branches and attached spikelets, caused incomplete panicle exsertion(Table 2), inhibited anther dehiscence and pollen shedding (Fig. 3), reduced pollen vigor (Fig. 4) and grain size (Fig. 1), especially under ADT, depending on the variety. Of the four rice varieties tested, SY63 displayed the highest tolerance to heat stress, whereas N22, HHZ, and LYPJ were heat-sensitive (Fig. 1). Yield reductions in heat-sensitive varieties were attributed primarily to reductions in spikelet fertility, spikelets per panicle, and grain weight (Figs 1 and 5).

The three high temperature treatments increased the contents of bound CTKs and ABA, and reduced the contents of active CTKs, GA_1_, and IAA (Table 3). The adverse effects of high temperature treatments were alleviated by application of exogenous 6-BA in a heat-susceptible variety (LYPJ). Heat-mediated phytohormone changes in young panicles affected yield by three possible mechanisms: (i) the reduced tZ + tZR levels disrupted the differentiation and promoted the degradation of secondary branches and attached florets, thereby reducing spikelets per panicle; (ii) the reduced IAA and GA_1_ levels caused panicle enclosure, reductions in pollen vigor and anther dehiscence, resulted in low spikelet fertility; and (iii) the reduced active CTKs, IAA, and GA_1_ resulted in reduced grain size and grain weight (Fig. 5). Noticeably, adverse effects of decreases in CTKs on panicle exsertion, spikelets per panicle, spikelet fertility, grain size and grain yield under high temperature treatments were relieved by application of exogenous 6-BA in the study, however, effects of IAA, GA_1_, and ABA on the traits under high temperature treatments were only supported by correlation analyses.

This study showed that heat-tolerant SY63 displayed better yield and yield components under high temperature treatments than the three heat-sensitive varieties. Generally, heat-treated SY63 maintained stable levels of IAA, GA_1_, tZ + tZR, and relatively high contents of iP + iPA + iPMP, active CTKs, and ABA in young panicles (Table 3), which generated complete panicle exsertion, stabilized the differentiated branches and florets, and protected against heat-induced reductions in pollen vigor, anther dehiscence, pollen shedding, and grain size (Table 2).

## Materials and methods

### Crop husbandry

Pot experiments were conducted between May and October 2013 at the experimental station of Huazhong Agricultural University, Wuhan City, Hubei Province, China (30°29′ N, 114°22′ E). Four rice varieties that have been extensively used in previous research on high temperature stress were used in this study: N22, HHZ, LYPJ, and SY63^3^,^12^.

The potted rice plants were randomly arranged with four replications under natural ambient conditions. Seeds were sown in plastic seeding trays with loam soil after breaking dormancy at 50 °C for five days. At the three-leaf stage, four seedlings of each variety were transplanted into a 14 L plastic pot (28.5 cm height × 30 cm top diameter × 25 cm bottom circumference) containing a mixture of 17 kg soil (loam:sand, 2:1) and 12.5 g compound fertilizer (N:P_2_O_5_:K_2_O, 16%:16%:16%). Seedlings were thinned to three plants per pot (each plant had three tillers) 8 days after transplanting, and the main tillers were tagged. A total of 1.0 g urea was topdressed per pot 10 days after transplanting. All pots were maintained in approximately 2 cm of water from sowing to maturity. Each pot was manually rotated by 90 degrees clockwise every seven days to avoid positional effects. Pests, diseases, birds, and weeds were intensively controlled.

### High temperature treatments

The facility used for high temperature treatments contains four individual greenhouses (4 m length × 4 m width × 4.5 m height). Each greenhouse is equipped with an air conditioner, a wetting machine, two ventilators, and two sensors for monitoring temperature and relative humidity. All greenhouses and equipment were connected to a central auto-controlled system (Auto-Greenhouse Monitoring and Data Management System, Version 3.00, Auto, China).

**Figure 6:**
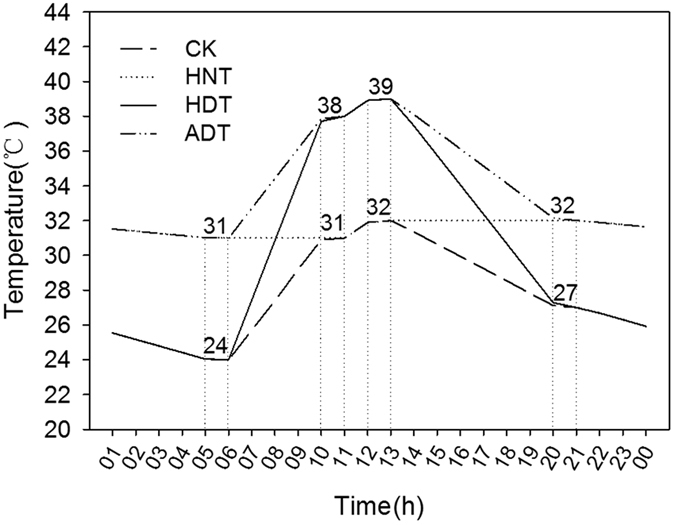
Temperature settings for three high-temperature treatments and one control. ADT, high whole-day temperature treatment; CK, control; HDT, high daytime temperature treatment; HNT, high nighttime temperature treatment.

The four temperature treatments were designated as high nighttime temperature from 19.00–07.00 hr (HNT), high daytime temperature from 07.00–19.00 hr (HDT), high whole-day temperature (ADT), and the control (CK). For CK treatment, temperatures were set at 24 °C during 05.00–06.00 hr, 31 °C during 10.00–11.00 hr, 32 °C during 12.00–13.00 hr, and 27 °C during 20.00–21.00 hr (Fig. 6). For HNT treatment, temperatures were set at 31 °C during 05.00–06.00 hr and 32 °C during 20.00–21.00 hr. For HDT treatment, temperatures were set at 38 °C during 10.00–11.00 hr and 39 °C during 12.00–13.00 hr. The ADT treatment used high temperature during all four time intervals (i.e., 31 °C during 05.00–06.00 hr, 38 °C during 10.00–11.00 hr, 39 °C during 12.00–13.00 hr, and 32 °C during 20.00–21.00 hr). Relative humidity was set at approximately 80%. The air temperature in the greenhouse was controlled by a central auto-controller, and gradually approached the next set temperature. Air temperature and relative humidity were recorded 5 cm above the canopy in the greenhouse using a stand-alone sensor (HOBO, H08-003-02, Onset Computer Corporation, Bourne, MA, USA).

The rice plants were moved to the controlled facilities on the approximate date of the start of panicle initiation determined by apical dissection, on which panicle emergence could be visually observed. Plants were treated with the high temperature regimens for 15 days, which corresponded approximately to stages R0–R2 according to Counce *et al*.^58^. The panicle lengths reached approximately 1.5–2.0 cm after 15 days of high temperature treatment. Plants were grown under naturally ambient conditions during all other growth stages before and after the high temperature treatments.

### Exogenous 6-benzylaminopurine treatments

The 6-BA solution (60 mg L^−1^) contained a total of 60 mg 6-BA (Sigma-Aldrich, USA) dissolved in 1 ml of 1% (w/v) NaOH, which was then diluted to 1 L with double-distilled H_2_O. Two drops of 0.01% (v/v) Tween 20 was added as a surfactant and mixed thoroughly. The 6-BA solution was sprayed onto stems under ADT in LYPJ and SY63, with 20 ml per dose per plant. The 6-BA solution was applied twice, one day before high temperature treatments and on the second day after high temperature treatments.

### Determination of differentiated and degraded branches and florets

For each experimental replicate, three main tillers from three pots (approximately half of these had exserted out from the flag leaf sheath) were collected from the control and the three high temperature treatment groups. Vestiges or small protrusions remaining on the panicle were considered as branches or florets that had degraded during panicle development^8^. The following two characters were counted and recorded: existing and aborted primary and secondary branches, and existing and degraded florets on primary and secondary branches. The total number of differentiated branches or florets that were recorded per panicle included the existing and retrograded branches or florets.

### Observation of panicle enclosure, anther dehiscence, and pollen vigor

Panicle enclosure is defined as the lower portion of a panicle that remains surrounded by the enclosing sheath of the flag leaf^42^. We determined the distance from the panicle neck node to the flag leaf auricle (designated as the length of the enclosed panicle part), and the distance from the panicle neck node to the panicle tip (designated as the full panicle length) of incompletely exserted panicles from the main tiller at maturity^39^. The enclosed panicle length of completely exserted panicles was designated as 0. Three main tillers were analyzed for each experimental replicate. The lengths of exserted panicles were reported in this study.

Anthers were collected at flowering by scraping the opening florets with a glass slide during 09.00–10.00 hr, and 30 anthers were retained on each slide. The collection was repeated three times for each sampling. Anthers with opened apical and/or basal pores were recorded as dehisced. Anther dehiscence percentage (%) was calculated as the ratio of dehisced anthers to total anthers.

Pollen grains were scattered on a slide with one drop of 1% iodine potassium iodide solution by gently tapping the opening florets, and anthers on the slide that had not dehisced were manually squeezed to release pollen grains^59^. The iodine-stained pollen grains were examined at 40× magnification using a DP70 digital camera attached to an Axioplan 2 microscope (Carl Zeiss, Jena, Germany). Three observations were performed per slide; each observation covered approximately 100 pollen grains. Pollen grains that were stained black and had spherical shape were considered as vigorous. The percentage of vigorous pollen grains (%) was calculated as the ratio of the vigorous pollen grains to total pollen grains.

### Determination of grain yield, yield components, and grain shape

At maturity, three main tillers for each experimental replicate were harvested and manually threshed to determine grain yield and yield components. Salt water (1.12 g cm^−3^ density) was used to separate filled grains from empty and unfilled grains. The grains were dried, and partially filled grains were picked out carefully by manually pressing the grains between the forefinger and thumb. Filled grains were oven dried at 80 °C to a constant weight to determine grain yield (g per panicle) and grain weight (mg per grain). The percentage of grain filling was calculated as the number of filled grains divided by the total number of grains per panicle. Spikelet fertility (%) was calculated as the number of filled and partially filled grains divided by the total number of grains per panicle.

Grain shape traits, including grain length (mm), grain width (mm), and grain area (mm^2^), were measured using the WSeen Measuring System for Rice Grain Quality (Hangzhou WSeen Detection Technology Co. Ltd., China) based on images captured by ScanMaker i800 (Microtek, Shanghai, China).

### Extraction and determination of phytohormones

At the end of the high temperature treatment, three young panicles from the main tillers of each experimental replicate were collected and frozen in liquid nitrogen, and then stored at −80 °C for phytohormone measurements. Phytohormones were extracted and purified according to the method of Xie and Zhang^60^. Phytohormones were quantified using high-performance liquid chromatography (HPLC) according to the method of Chou *et al*.^50^, with minor modifications.

Briefly, frozen panicles were cut into small pieces and completely mixed. Then, 1 g of tissue was ground in a chilled mortar on ice with 8 ml 75% methanol containing 5% formic acid in 20% double-distilled H_2_O. The homogenates were transferred to 10 ml centrifuge tubes, incubated at 4 °C for 12 h, centrifuged at 12,000 × *g* for 20 min at 4 °C, and supernatants were collected. The pellets were subjected to phytohormone extraction two more times as described above (e.g., 8 ml of 75% alcohol containing 5% formic acid in 20% double-distilled H_2_O and centrifugation). All supernatants were pooled and condensed to 2 ml using a freeze dryer. Then, petroleum ether was used for three consecutive extractions of pigments and phenolics. The petroleum ether containing pigments and phenolics was removed, the lower phase was freeze dried, and the sample was resuspended in 3 ml of sodium acetate (1 mol/L, pH 8.0). This solution is designated as the crude extracts, which was used for further analyses of specific phytohormones.

The crude extracts were extracted three times with 1-butanol, and the upper phases containing CTKs were collected. The lower phases were adjusted to pH 3.0 and extracted three more times with ethyl acetate. The upper phases containing IAA, GAs, and ABA were collected. All these collected phases containing CTKs, IAA, GAs, and ABA were pooled and freeze dried. Finally, the freeze dried samples were dissolved in 5 ml methyl alcohol and purified using a C_18_-SepPak cartridge (Waters Corporation, Milford, MA, USA). The purified samples were freeze dried, and then dissolved in 0.8 ml methyl alcohol for phytohormone determination. Phytohormones were measured using HPLC with a multi-step linear gradient elution (45 min) at a flow rate of 1.6 ml min^−1^ under constant column temperature at 45 °C with UV detection at 269 nm.

The calibration standards included a mixed phytohormone standard solution containing tZ9G, tZ, dihydrozeatin, tZR, dihydrozeatin riboside, iPMP, iP, iPA, iP9G, and GA_1_ (OlChemIm Ltd., Czech Republic) and ABA and IAA standards (Sigma, St. Louis, USA). The calibration standards were prepared at concentrations of 5.7, 8.5, 11.4, 45.6, 91.1, and 182.3 ng mL^−1^ for each hormone standard in the mixed standard solution of 12 compounds. Calibration standard curves were repeated four times, and then a standard curve for each compound was calculated.

To evaluate the recovery of phytohormone extraction, another 1 g sample of panicle tissue was ground with 150 ng of each hormone standard defined above. The percentage recovery of tZ9G, tZ, dihydrozeatin, tZR, dihydrozeatin riboside, iPMP, iP, iPA, iP9G, GA_1_, ABA, and IAA was 90.9 ± 1.8, 109.4 ± 5.4, 102.2 ± 5.1, 97.8 ± 4.4, 99.1 ± 4.6, 62.1 ± 2.2, 96.7 ± 4.1, 97.2 ± 5.0, 96.7 ± 4.7, 93.9 ± 1.9, 99.3 ± 5.1, and 82.5 ± 4.5%, respectively.

The phytohormones dihydrozeatin and dihydrozeatin riboside were not detected in young panicles in this study. Bound CTKs included tZ9G and iP9G as inactive CTKs. The other CTKs (tZ, tZR, iP, iPA, and iPMP) were designated as active CTKs. The content of each phytohormone was calculated based on the derived standard curves, and expressed in units of ng per g fresh weight (FW).

### Statistical analysis

The means of four experimental replicates were used for analysis. We performed analysis of variance (ANOVA) first using Statistix 8.0 (Analytical Software, Tallahassee, Florida, USA). The ANOVA showed that variance due to temperature treatments and varieties for each investigated trait are significant. Then, differences among means were compared by the least significant difference (LSD) test at *P* < 0.05.

The change (Δ) in a certain trait was calculated as: Δ = Value_HT_ – Value_CK_. Positive Δ indicates an increase, and negative Δ indicates a decrease induced by high temperature treatments. To measure the association among changes in phytohormones and panicle traits, Pearson correlation analysis of the mean changes (Δ) in the investigated traits was used across four varieties and three high temperature treatments (*n* = 12) using SPSS 22.0 (SPSS Inc., Chicago, IL). The significance of correlation coefficient was tested using Student’s t-test (two-tailed). Additionally, under the hypothesis that *ρ* = 0, a t-test was performed to determine whether the correlation coefficient is significantly different from zero.

The percentage change was calculated according to the following equation: percentage (%) = (Value_HT_ – Value_CK_)/ Value_CK_×100%, where Value_HT_ is the value under high temperature treatment and Value_CK_ is the value in the control. The relative value was defined as the ratio of the value under high temperature treatment to that of the control.

## Additional Information

**How to cite this article**: Wu, C. *et al*. Heat-induced phytohormone changes are associated with disrupted early reproductive development and reduced yield in rice. *Sci. Rep*. **6**, 34978; doi: 10.1038/srep34978 (2016).

## Figures and Tables

**Table 1:** Maximum, minimum and average daytime and nighttime temperatures.

Treatment group	Daytime temperature (°C)			Nighttime temperature (°C)		
	Maximum temperature	Minimum temperature	Average temperature	Maximum temperature	Minimum temperature	Average temperature
CK	37.3	25.1	31.9	33.4	22.7	27.2
HNT	42.6	23.3	33.5	39.5	20.6	31.9
HDT	45.6	24.5	36.1	36.8	20.0	26.7
ADT	45.9	21.7	38.3	36.9	28.8	31.5

ADT, high whole-day temperature treatment; CK, control; HDT, high daytime temperature treatment; HNT, high nighttime temperature treatment.

**Table 2:** High temperature treatment effects on panicle-related traits.

Variety	Treat- ment group	PB	SB	RSB	FPB	FSB	RFPB	RFSB	Exserted panicle length (cm)	Anther dehiscence (%)	Vigorous pollen (%)	Grain length (mm)	Grain width (mm)	Grain area (mm^2^)
N22	CK	11 ± 0.3^a^	35 ± 1.6^a^	13 ± 0.6^b^	63 ± 0.7^a^	69 ± 2.6^a^	1 ± 0.2^a^	3 ± 0.7^c^	20.3 ± 1.4^a^	94.5 ± 1.8^a^	91.8 ± 6.3^a^	7.6 ± 0.1^a^	2.7 ± 0.0^a^	14.8 ± 0.2^a^
	HNT	11 ± 0.2^a^	35 ± 1.0^a^	13 ± 1.3^b^	62 ± 1.5^a^	68 ± 1.5^a^	1 ± 0.2^a^	4 ± 0.8^c^	16.1 ± 1.2^b^	92.4 ± 2.4^a^	88.0 ± 8.0^a^	7.4 ± 0.1^b^	2.6 ± 0.1^ab^	14.6 ± 0.3^a^
	HDT	10 ± 0.6^a^	35 ± 0.8^a^	13 ± 0.7^b^	63 ± 1.4^a^	68 ± 2.3^a^	1 ± 0.2^a^	5 ± 0.9^b^	16.2 ± 1.3^b^	92.4 ± 0.9^a^	85.8 ± 5.5^a^	7.4 ± 0.1^bc^	2.5 ± 0.1^b^	13.6 ± 0.3^b^
	ADT	10 ± 0.7^a^	30 ± 2.1^b^	16 ± 1.1^a^	62 ± 1.7^a^	60 ± 3.2^b^	1 ± 0.9^a^	10 ± 0.2^a^	6.3 ± 1.0^c^	90.5 ± 5.0^a^	82.8 ± 7.6^a^	7.2 ± 0.1^c^	2.3 ± 0.2^c^	12.8 ± 0.7^c^
HHZ	CK	14 ± 0.4^a^	61 ± 1.3^a^	22 ± 1.3^b^	78 ± 4.1^a^	118 ± 3.5^a^	0 ± 0.0^b^	3 ± 0.4^c^	22.2 ± 0.7^a^	87.8 ± 1.8^a^	89.0 ± 5.4^a^	9.0 ± 0.1^a^	2.2 ± 0.0^a^	14.9 ± 0.1^a^
	HNT	13 ± 1.0^a^	50 ± 1.4^c^	23 ± 0.9^b^	78 ± ± 4.0^a^	107 ± 2.7^bc^	4 ± 2.8^a^	7 ± 0.5^b^	17.2 ± 1.1^c^	85.3 ± 2.4^ab^	55.5 ± 8.8^c^	8.8 ± 0.4^b^	2.2 ± 0.1^a^	14.7 ± 0.9^a^
	HDT	14 ± 1.3^a^	54 ± 0.7^b^	23 ± 1.1^b^	78 ± 3.1^a^	109 ± 4.4^b^	1 ± 0.5^b^	7 ± 1.0^b^	20.4 ± 0.9^b^	85.7 ± 0.7^ab^	68.9 ± 6.4^b^	8.5 ± 0.2^c^	2.1 ± 0.0^ab^	13.7 ± 0.4^b^
	ADT	13 ± 0.3^a^	49 ± 1.0^c^	26 ± 1.0^a^	78 ± 1.9^a^	102 ± 3.9^c^	2 ± 1.7^ab^	12 ± 1.2^a^	15.4 ± 13.4^d^	83.8 ± 1.9^b^	40.2 ± 6.5^d^	8.6 ± 0.2^c^	2.1 ± 0.1^b^	13.7 ± 0.4^b^
LYPJ	CK	13 ± 0.3^a^	54 ± 0.6^a^	15 ± 1.7^c^	75 ± 1.1^a^	121 ± 5.6^a^	0 ± 0.0^b^	5 ± 0.5^d^	21.7 ± 0.4^a^	89.1 ± 1.1^a^	91.0 ± 7.5^a^	9.2 ± 0.1^a^	2.6 ± 0.1^a^	17.7 ± 0.4^a^
	HNT	13 ± 0.3^a^	48 ± 1.1^b^	23 ± 0.3^a^	71 ± 5.4^a^	88 ± 3.6^b^	3 ± 0.3^a^	8 ± 0.8^c^	20.2 ± 1.5^ab^	84.4 ± 2.0^bc^	57.5 ± 7.0^c^	9.0 ± 0.0^ab^	2.6 ± 0.1^a^	17.4 ± 1.1^a^
	HDT	13 ± 0.8^a^	48 ± 1.1^b^	24 ± 1.4^a^	75 ± 4.8^a^	85 ± 4.0^b^	0 ± 0.0^b^	13 ± 1.7^b^	19.5 ± 0.4^b^	86.3 ± 2.3^ab^	76.7 ± 7.4^b^	8.6 ± 0.1^cd^	2.6 ± 0.1^a^	17.1 ± 0.8^a^
	ADT	13 ± 0.4^a^	45 ± 1.0^c^	24 ± 1.3^a^	68 ± 5.7^a^	74 ± 11.7^c^	0 ± 0.0^b^	17 ± 1.6^a^	16.9 ± 1.3^c^	82.1 ± 0.4^c^	40.8 ± 6.1^d^	8.6 ± 0.3^d^	2.6 ± 0.0^a^	16.8 ± 0.7^a^
	ADT-6BA	13 ± 0.7^a^	54 ± 2.3^a^	21 ± 1.5^b^	74 ± 3.1^a^	88 ± 2.9^b^	0 ± 0.0^b^	9 ± 1.5^c^	21.5 ± 1.0^a^	—	—	8.9 ± 0.1^bc^	2.5 ± 0.1^a^	17.0 ± 0.3^a^
SY63	CK	12 ± 1.2^a^	40 ± 1.4^a^	20 ± 0.3^a^	71 ± 2.9^a^	64 ± 1.9^a^	0 ± 0.0^a^	2 ± 0.3^a^	24.1 ± 0.9^a^	87.7 ± 1.7^a^	92.8 ± 7.3^a^	8.4 ± 0.1^ab^	2.9 ± 0.1^a^	18.4 ± 0.4^a^
	HNT	12 ± 0.5^a^	41 ± 1.6^a^	20 ± 0.6^a^	72 ± 1.2^a^	64 ± 1.9^a^	0 ± 0.0^a^	3 ± 1.0^a^	24.3 ± 2.3^a^	87.6 ± 2.2^a^	90.9 ± 5.8^a^	8.5 ± 0.1^a^	2.8 ± 0.1^a^	18.6 ± 0.6^a^
	HDT	12 ± 0.4^a^	42 ± 0.7^a^	21 ± 1.5^a^	70 ± 1.4^a^	62 ± 4.0^a^	0 ± 0.0^a^	3 ± 0.3^a^	23.8 ± 0.5^a^	86.4 ± 3.1^a^	91.9 ± 7.8^a^	8.2 ± 0.1^c^	2.8 ± 0.0^ab^	17.6 ± 0.4^b^
	ADT	12 ± 0.5^a^	42 ± 1.5^a^	21 ± 2.4^a^	71 ± 1.4^a^	62 ± 2.3^a^	0 ± 0.0^a^	3 ± 1.6^a^	23.8 ± 1.1^a^	85.8 ± 0.6^a^	88.4 ± 6.0^a^	8.3 ± 0.1^bc^	2.7 ± 0.0^b^	17.5 ± 0.3^b^
	ADT-6BA	12 ± 0.5^a^	41 ± 3.2^a^	21 ± 2.8^a^	71 ± 1.0^a^	64 ± 1.5^a^	0 ± 0.0^a^	3 ± 0.4^a^	22.7 ± 1.3^a^	—	—	8.3 ± 0.1^bc^	2.7 ± 0.0^b^	17.5 ± 0.4^b^

Data are presented as mean ± SD (*n* = 4). Different letters within a column indicate statistical differences among the four temperature treatments for each variety at the *P* < 0.05 level by the least significant difference (LSD) test. ADT, high whole-day temperature treatment; CK, control (natural ambient temperature); FPB, number of differentiated florets on primary branches; FSB, number of differentiated florets on secondary branches; HDT, high daytime temperature treatment; HHZ, Huanghuazhan; HNT, high nighttime temperature treatment; LYPJ, Liangyoupeijiu; N22, Nagina22; PB, number of differentiated primary branches; RFPB, number of retrograded florets on primary branches; RFSB, number of retrograded florets on secondary branches; RSB, number of retrograded secondary branches; SB, number of differentiated secondary branches; SY63, Shanyou 63.

**Table 3:** High temperature treatment effects on phytohormone concentrations (ng/g) in panicles.

Variety	Treatment group	CTKs	aCTKs	tZ9G + iP9G	tZ + tZR	iP + iPA + iPMP	GA_1_	IAA	ABA
N22	CK	482 ± 16.1^a^	336 ± 17.0^a^	145 ± 9.4^c^	114 ± 1.9^a^	223 ± 16.1^a^	107 ± 6.8^a^	134 ± 6.5^a^	87 ± 5.8^b^
	HNT	484 ± 36.1^a^	268 ± 17.1^b^	216 ± 21.9^b^	116 ± 9.6^a^	152 ± 9.0^c^	59 ± 7.1^b^	44 ± 4.6^b^	94 ± 5.1^ab^
	HDT	486 ± 7.4^a^	270 ± 7.8^b^	216 ± 11.7^b^	100 ± 5.1^b^	170 ± 11.7^b^	54 ± 5.8^b^	40 ± 2.9^b^	105 ± 7.6^a^
	ADT	439 ± 25.6^b^	152 ± 4.2^c^	287 ± 21.9^a^	80 ± 6.2^c^	72 ± 2.6^d^	34 ± 4.9^c^	17 ± 1.7^c^	109 ± 15.3^a^
HHZ	CK	573 ± 30.8^a^	376 ± 11.3^a^	197 ± 9.1^c^	169 ± 9.3^a^	208 ± 3.5^a^	86 ± 3.2^a^	123 ± 6.1^a^	58 ± 3.9^a^
	HNT	561 ± 38.9^a^	260 ± 13.2^b^	301 ± 33.0^b^	129 ± 4.7^b^	131 ± 11.3^b^	66 ± 4.8^b^	42 ± 3.6^c^	66 ± 6.0^a^
	HDT	529 ± 10.3^a^	213 ± 14.3^c^	316 ± 7.3^b^	142 ± 12.5^b^	71 ± 3.6^c^	73 ± 6.4^b^	78 ± 7.0^b^	66 ± 5.3^a^
	ADT	531 ± 39.6^a^	168 ± 9.9^d^	362 ± 31.8^a^	112 ± 10.3^c^	56 ± 1.6^d^	43 ± 3.2^c^	30 ± 3.6^d^	67 ± 6.3^a^
LYPJ	CK	709 ± 25.4^a^	441 ± 25.6^a^	268 ± 9.9^b^	184 ± 14.6^a^	257 ± 13.8^a^	104 ± 6.1^a^	200 ± 8.6^a^	86 ± 4.4^a^
	HNT	649 ± 32.8^b^	329 ± 14.6^b^	320 ± 18.5^a^	145 ± 14.0^b^	184 ± 5.1^b^	74 ± 9.3^c^	68 ± 3.1^c^	96 ± 1.2^a^
	HDT	618 ± 15.3^b^	285 ± 10.3^c^	333 ± 10.2^a^	135 ± 11.0^b^	150 ± 5.6^c^	93 ± 7.9^b^	136 ± 5.5^b^	97 ± 12.4^a^
	ADT	544 ± 37.4^c^	189 ± 10.7^d^	355 ± 44.8^a^	112 ± 9.0^c^	77 ± 2.5^d^	60 ± 4.5^d^	35 ± 2.8^d^	97 ± 6.8^a^
SY63	CK	590 ± 25.1^a^	353 ± 11.6^a^	238 ± 17.3^a^	114 ± 6.2^a^	238 ± 8.5^a^	86 ± 6.8^a^	111 ± 9.4^a^	69 ± 5.2^b^
	HNT	566 ± 25.4^a^	318 ± 21.9^b^	249 ± 14.9^a^	116 ± 4.7^a^	201 ± 20.3^b^	91 ± 8.5^a^	115 ± 12.0^a^	93 ± 8.4^a^
	HDT	568 ± 33.2^a^	316 ± 19.8^b^	252 ± 14.4^a^	114 ± 9.9^a^	201 ± 14.2^b^	88 ± 7.8^a^	109 ± 8.2^a^	97 ± 7.7^a^
	ADT	549 ± 38.8^a^	296 ± 30.6^b^	253 ± 24.3^a^	111 ± 8.7^a^	182 ± 32.6^b^	86 ± 10.5^a^	112 ± 8.8^a^	97 ± 9.7^a^

Data are presented as mean ± SD (*n* = 4). Different letters within a column indicate statistical differences among the four temperature treatments for each variety at the *P* < 0.05 level by the least significant difference (LSD) test. aCTKs, active cytokinin compounds (tZ + tZR + iP + iPA + iPMP); ADT, high whole-day temperature treatment; CK, control (natural ambient temperature); CTKs, total cytokinins; HDT, high daytime temperature treatment; HHZ, Huanghuazhan; HNT, high nighttime temperature treatment; LYPJ, Liangyoupeijiu; N22, Nagina22; SY63, Shanyou 63.

**Table 4:** Pearson correlation coefficients among changes in phytohormones and changes in panicle traits and yield components across different varieties and heat treatments (*n* = 12).

Phytohormone changes	ΔYield	ΔSpikelets /panicle	ΔPB	ΔSB	ΔRSB	ΔFPB	ΔFSB	ΔRFPB	ΔRFSB	ΔSpikelet fertility	ΔExserted panicle length	ΔAnther dehiscence	ΔVigorous pollen	ΔGrain weight	ΔGrain length	ΔGrain width	ΔGrain area
ΔiP9G + tZ9G	−0.82**	−0.67*	−0.66*	−0.80**	0.16	0.03	−0.20	0.28	0.67*	−0.70*	−0.77**	−0.48	−0.58*	−0.77**	−0.49	−0.34	−0.55
ΔtZ + tZR	0.87**	0.92**	0.57	0.87**	−0.80**	0.52	0.86**	−0.35	−0.96**	0.40	0.40	0.84**	0.88**	0.86**	0.68*	−0.26	0.22
ΔiP + iPA + iPMP	0.76**	0.74**	0.33	0.73**	−0.54	0.41	0.60*	0.02	−0.90**	0.53	0.61*	0.77**	0.70*	0.89**	0.78**	0.12	0.54
ΔaCTKs	0.83**	0.84**	0.43	0.8**	−0.66*	0.46	0.71**	−0.11	−0.96**	0.51	0.56	0.83**	0.78**	0.92**	0.78**	−0.01	0.45
ΔCTKs	0.40	0.57	−0.05	0.40	−0.84**	0.75**	0.89**	0.13	−0.77**	0.02	0.04	0.77**	0.58*	0.59*	0.67*	−0.40	0.10
ΔGA_1_	0.51	0.17	0.35	0.29	−0.14	0.29	0.16	0.02	−0.40	0.98**	0.85**	0.56	0.26	0.60*	0.33	0.65*	0.50
ΔIAA	0.75**	0.53	0.49	0.56	−0.60*	0.67*	0.66*	−0.27	−0.65*	0.79**	0.58*	0.88**	0.66*	0.72**	0.49	0.10	0.17
ΔABA	0.72**	0.69*	0.57	0.74**	−0.42	0.17	0.51	−0.50	−0.51	0.31	0.17	0.46	0.66*	0.43	0.42	−0.33	−0.19

^*^And ^**^indicate significant Pearson correlation at the *P* < 0.05 and *P* < 0.01 level (two-tailed), respectively. Δ, change in trait was defined as the difference of the value under high temperature treatment from the value under the control. FPB, number of differentiated florets on primary branches; FSB, number of differentiated florets on secondary branches; PB, number of differentiated primary branches; RFPB, number of retrograded florets on primary branches; RFSB, number of retrograded florets on secondary branches; RSB, number of retrograded secondary branches; SB, number of differentiated secondary branches.
